# Supplementary material for: The base of the Lystrosaurus Assemblage Zone, Karoo Basin, predates the end-Permian marine extinction
Source: Nat Commun. 2020 Mar 18;11:1428. doi: 10.1038/s41467-020-15243-7 (PMC7080820; doi:10.1038/s41467-020-15243-7)
Supplement: Supplementary file 1 — Supplementary Information [file 41467_2020_15243_MOESM1_ESM.pdf]

The base of the *Lystrosaurus* Assemblage Zone, Karoo Basin, predates the end-Permian marine extinction

Gastaldo et al.

Table of Contents

Supplementary Note 1: Lithostratigraphy and sampling
Supplementary Note 2: Magnetostratigraphy and comparison with the geomagnetic polarity time scale
Supplementary Note 3: Mercury
Supplementary Note 4: Palynology
Assemblages
Age assignment
Supplementary References
Supplementary Figure 1. Locality maps.
Supplementary Figure 2. Measured stratigraphic section
Supplementary Figure 3. Petrographic images of the ash fall deposit and host hematitic mudstone sampled at site 2950–2963 m in the Nooitgedacht section for high precision U-Pb zircon geochronology.
Supplementary Figure 4. Paleomagnetic results from thinly bedded hematitic siltstone/mudstone collected from stratigraphic intervals within five centimeters above and below the ash fall deposit sampled for U-Pb zircon high precision geochronology.
Supplementary Figure 5. Paleomagnetic results from thinly bedded hematitic siltstone/mudstone beds collected from additional stratigraphic intervals within five meters above and below the ash fall deposit sampled for U-Pb zircon high precision geochronology.
Supplementary Figure 6. Results of continuous bulk susceptibility measurements as a function of heating from room temperature to 685° C and cooling back to room temperature.
Supplementary Figure 7. Estimates of the geomagnetic polarity time scale from the late Permian to earliest Triassic, modified from (A) Szurlies and (B) Hounslow and Balabanov.
Supplementary Figure 8. Representative palynomorphs recovered from greenish-gray coarse siltstone at a stratigraphic height of 29.9 m (see supplemental Fig. 1; UCMP collection locality number PA1378.02).
Supplementary Figure 9. Representative palynomorphs recovered from greenish-gray coarse siltstone at a stratigraphic height of 40.42 m (see supplemental Fig. 1; UCMP collection locality number PA1378.01).

Supplementary Information

The base of the *Lystrosaurus* Assemblage Zone, Karoo Basin, predates the end-Permian marine extinction

Supplementary Note 1: Lithostratigraphy and sampling

The stratigraphy of farm Nooitgedacht (Supplementary Figure 1) is similar to other Changhsingian successions in the Karoo and consists of a seemingly monotonous succession of fine to very-fine grained feldspathic wacke, greenish gray (5GY 6/1), grayish brown (5 YR 4/2) and mottled mudrock, arranged in upwards-fining cycles (Supplementary Figure 2). Mudrock intervals are lateral to, below and above, sandstone bodies, and upsection display increasingly reddish and brownish colors, while isolated and amalgamated sandstone lenses increase in abundance. Similar to what has been documented elsewhere(1, n) these sandstones thin and split laterally, grading into "heterolithic beds" consisting of thinly bedded, reddish gray and olive gray lenses of (coarse) siltstone; some of which have been associated with the *Daptocephalus* – *Lystrosaurus* AZ boundary and "PTB"(3, 4, but see 2). Overlying the interval identified as the vertebrate-defined Permian–Triassic Boundary (PTB)(5) is a succession characterized by dusky red and olive-gray bedded mudrock that grades into a sheet-sandstone succession. This sheet-sandstone interval (Fig. 2c) consists of stacked, fining-up cycles, bounded by fine- to very-fine grained feldspathic wacke in which intraformational conglomerate lags occur infrequently(6), and separated by thick siltstone intervals. This section was measured using standard field practice and a 1.5 m Jacob Staff with Abney level, following the protocols of Gastaldo et al.(8-10).

About 12 m above the vertebrate-defined PTB in our section, a thick sheet sandstone represents the base of the first of several fining-up cycles (Supplementary Figure 2a). It is in the dusky red and olive-gray mudrock interval, above the proposed "PTB" of Botha-Brink et al.(5)

and below the first thick channel-fill, where a thin (up to ~1.0 cm thick) ash bed crops out (Supplementary Figure 2b, yellow arrow), and from which we have recovered a population of pristine, euhedral zircon grains (Supplementary Figure 3). While it appear massive and predominantly dusky red in outcrop (Fig. 2a), the mudrock of this interval consists of stacked upwards-fining beds of fine to coarse, dusky red and olive-gray siltstone (Supplementary Figure 2b). Internally these beds exhibit massive bedding, although faint cross-lamination are observed in better cemented units .

Samples for magnetic polarity information were collected in thinly bedded siltstone within 5 cm above and below the ash bed, and also from additional horizons within five meters above and below the ash horizon (see below). Hand samples collected for TOC:Hg analyses were obtained from two intervals: the first spans 80 cm across the horizon identified by Botha-Brink et al.[(5)] as the vertebrate-defined PTB, the second spans 40 cm across the ash-fall interval. Six palynological samples were processed from siltstone beds above the air-fall ash, four (Supplementary Figure 2b, black **P**) yielded pollen-and-spore spectra, two of which are reported here because of sufficient yields.

Petrographic images of the ash-fall deposit sampled for high precision U-Pb zircon geochronology hosted by hematitic mudrock show textures that are consistent with a primary, ash fall origin with little post depositional reworking (Supplementary Figure 3). Supplementary Figure 3a is a scan of most of a large format, uncovered thin section prepared from a field-impregnated sample of the ash deposit and underlying mudstone. The scanned image was obtained using a GIGAmacro Magnify2 photo stand with an attached Canon Rebel T5i digital camera. Supplementary Figures 3a, 3b, and Sc are petrographic images of parts of the ash deposit. Supplementary Figure 3b is a transmitted light (uncrossed polars) photomicrograph of a

part of the ash bed (note scale bar); the area highlighted by a dotted yellow rectangle includes well-expressed cross-stratification features in the deposit. Supplementary Figure 3c is a transmitted light (uncrossed polars) photomicrograph of another part of the ash bed; arrows point to specific mineral fragments in the deposit (q-quartz, a-amphibole, b-biotite, p-plagioclase). Supplementary Figure 3d is a transmitted light (crossed polars) photomicrograph of another part of the ash bed (note scale bar); area highlighted by dotted yellow rectangle includes well-expressed cross-stratification features in the deposit.

Supplementary Note 2: Magnetic Polarity information and comparison with the geomagnetic polarity time scale

Hematitic thinly bedded siltstone/mudstone collected from stratigraphic intervals within five centimeters above and below the ash fall deposit sampled for U-Pb zircon high precision geochronology yield interpretable demagnetization behavior (Supplementary Figure 4). This behavior is also repeated from additional intervals in thinly bedded siltstones/mudstones sampled within about five meters above and below the ash fall deposit (Supplementary Figure 5, Supplementary Table 2). Supplementary Figure 4a shows images of oriented (with respect to geographic north and the vertical) hematitic mudstone fragments, sampled immediately above and below the ash-fall deposit, in ceramic cubes, without and with non-magnetic alumina cement. The ceramic cubes are 20 mm on a side. Supplementary Figure 4b shows equal area projections of the directions of magnetization (first-removed and higher laboratory unblocking temperature interval) vectors isolated in progressive thermal demagnetization. Supplementary Figures 4c and 4d provide examples of progressive thermal demagnetization results from specimens collected within 5 cm above (c) and below (d) the ash fall deposit. Orthogonal plots showing the endpoint of the magnetization vector plotted on the horizontal (solid circles) and vertical (open circles) planes. Also shown are equal area

projections of the remanence vector as a function of progressive demagnetization and normalized (to 300° C) plots of the remanence intensity as a function of progressive thermal demagnetization. Ceramic cube specimens collected from additional thinly bedded siltstones/mudstone intervals up to 5 meters above and below the ash fall deposit also show similar behavior in progressive demagnetization (Supplementary Figure 5) and yield similar directions of magnetization (Supplementary Table 2). Measurements of the bulk susceptibility of crushed fragments of representative thinly bedded siltstone/mudstone as a function of heating and subsequent cooling reveal the presence of low concentrations of both hematite and relatively subordinate magnetite, as there is a gradual decrease in susceptibility to the approximate Neel temperature of hematite (~680° C; Supplementary Figure 6). The heating/cooling curves are irreversible, with a substantial increase in susceptibility in cooling, with the apparent production of a magnetic phase with an inferred Curie temperature between about 570 and 530° C.

The geomagnetic polarity time scale for the time interval across the Permian-Triassic boundary has been the subject of considerable interest for several decades. Relatively recent compilations include those by Szurlies[11] and Hounslow and Balabanov[12] (Supplementary Figures 7a, 7b, respectively). In each of the compilations shown, the inferred ages shown (medium gray, italics font) are those given by the authors. Note that the age scale is different in each of the compilations. The age in red is the interpreted best estimate of the age of the PTB; the age in purple is our reported age for the ash deposit in the NGT section. The compilation by Szurlies[11] includes a long (~ 700 ka) normal polarity chron that includes the PTB, and that of Hounslow and Balabanov[12] places the PTB as essentially the base of a similar, but slightly shorter normal polarity chron. The normal polarity magnetozone in which the ash deposit lies can be interpreted to match the geomagnetic polarity time scale in two ways. The first would

place the magnetozone as the earliest part of the normal polarity chron that includes the PTB, which is consistent with the compilation by Szurlies[11]. Alternatively, the normal polarity magnetozone is part of the next older normal polarity chron in the late Changhsingian.

Supplementary Note 3: Mercury

Mercury spikes in marine records have been identified for all five major mass extinction events[13–18]. In general, they have been marked by ratios of Hg (ppb) / TOC (wt. %) of greater than 200, with TOC values above 0.1 wt. %. For the end-Permian extinction event, well-associated with Siberian Traps volcanism, marine records globally have recorded enriched ratios greater than 200[18] and at some sites > 1000[16, 19]. Such enrichments have also been identified in terrestrial environments such as the fluvio-lacustrine sediments in Astartekløft, Greenland, at the end-Triassic extinction event associated with CAMP volcanism. Here, ratios of greater than 500 ppb/%C marked the boundary zone [20]. Most recently, several Hg spikes have been identified in terrestrial deposits in China coinciding with the PTB[21]. Similar to excursions found in the marine record, Hg peaks also rose above 1000 Hg (ppb) / TOC (wt. %) in these deposits[21]. In the current study, the Hg (ppb) / TOC (wt. %) from siltstone sampled across the biozone boundary of Botha-Brink et al.[5] and the ash bed, record values below 100 (Fig. 2; Supplementary Table 2), with the lowest values (0.84) obtained directly at the biozone boundary. As such, these values would be considered background and do not provide evidence for mercury enrichment in the intervals at Nooitgedacht. The possibility exists that the Karoo Basin might be too remote from a potential Siberian source area. Yet, the global nature of Hg atmospheric deposition and the high levels found in the terrestrial environment in the recent southwestern China study[21] suggests that a global signal is likely.

Supplementary Note 4: Palynology

Assemblages – The palynomorph assemblage at a stratigraphic height of 29.9 m (Supplementary Figure 8) is of low diversity and dominated by algal remains and simple spores. The dominant algal remains are *Leiosphaeridia* sp., less common are *Micrhystridium* and *Brazilea*. Common are simple trilete spores, some of which can be identified as *Brevitriletes cornutus*, *Altitriletes* sp. cf. *A. densus,* and *Punctatisporites gretensis*. Cingulate or cavate spores are not present. Most bisaccates have detached sacci and, except for very few taeniate specimens (*Lunatisporites* sp. cf. *L. pellucidus* and *Protohaploxypinus* sp. cf. *P. samoilovichii*), difficult to differentiate even at the generic level. The only observed monosulcate pollen in the assemblage is cf. *Cycadopites cymbatus*.

The palynomorph assemblage at a stratigraphic height of 40.42 m (Supplementary Figure 9) is more diverse than the older assemblage and dominated by algal remains, with simple spores and sulcate pollen being the dominant terrestrial components. *Leiosphaeridia* sp. is the dominant type of algal remains; *Brazilea* sp., *Quadrisporites horridus,* and *Reticulatisporites pseudopalliatus* are common; *Rugaletes playfordii*, *Micrhystridium* sp., and *Mehlisphaeridium fibratum* are rare. The most common simple trilete spores are *Horriditriletes* sp. cf. *H. tereteangulatus*, *Horriditriletes filiformis*, and *Brevitriletes cornutus*, with *Punctatisporites priscus*, *Leiotriletes directus*, *Osmundacidites wellmanii*, and *Lophotriletes* sp. cf. *L. novicus* being less abundant. Other spores are the cingulate *Limatulasporites* sp., the cavate *Densoisporites nejburgii,* and a large (potential) megaspore. The bisacates consist of rare taeniate forms that resemble *Lueckisporites virkkiae* and *Lunatisporites*, and the non-taeniate *Falcisporites stabilis*. Polyplicate and monosulcate pollen are represented by cf. *Ephedripites* sp. and *Cycadopites cymbatus*.

Botanical affinities – Most acavate trilete spores, such as *Horriditriletes*, *Osmundacidites*, and *Lophotriletes*, are characteristic of ferns[22]. *Leiotriletes* and *Punctatisporites* are, however, produced by a wide range of plant taxa[22]. The cavate spore *Densoisporites nejburgii* is produced by Pleuromeiaceae, a group of cormose heterosporous lycopods[23, 24]. The parent plant of the cingulate *Limatulasporites* is unknown. Pollen genera with many taeniae, such as *Protohaploxypinus*, have been been found *in situ* in the pollen sac clusters *Arberiella*. These structures are identical to those produced by several glossopterid microsporophylls[25, 26], and pollen sacs attached to the microsporophyll *Eretmonia*[27]. Taeniate bisaccates *Lueckisporites* and *Lunatisporites* were likely produced by conifers[28, 29]. In the southern hemisphere, *Falcisporites* can represent both peltasperms and corystosperms. This alete bisaccate genus is associated with the peltasperm *Lepidopteris*, which is known from Lower Triassic sections in Australia and Antarctica[26, 30]. *Falcisporites* has also been found *in situ* in the *Dicroidium*-attributed pollen organ *Pteruchus*[31, 32]. This corystosperm seed fern taxon was recently also discovered in putative upper Permian strata of Jordan[33, 34] and India[35], but is better known from Triassic Gondwanan floras (including South Africa). The pollen *Ephedripites* and *Cycadopites* likely represent peltasperms[22].

Age assignment – The Australian palynostratigraphic biozones are the standard for dating terrestrial sections of Lopingian to Early Triassic age in the southern hemisphere. These biozones are among the few that are calibrated against both marine invertebrate zones and U–Pb zircon dates[36–38]. The pollen-and-spore biozones are mostly based on either the first, or consistent occurrence of indicator taxa in basins in western[39] and eastern Australian[40]. The start of a major compositional change in the palynological assemblages occurs at the transition between the *Dulhuntyispora parvithola* and the *Playfordia crenulata*[37, 38]. This change entails a

transition from assemblages characterized by highly diverse multitaeniate bisaccates to ones dominated by algal remains, and relatively low amounts of alete non-taeniate bisaccates, cavate spores, and few multitaeniate bisaccate taxa. High resolution records show that this decline in number and abundance of specific multitaeniate bisaccate pollen taxa (e.g., *Protohaploxypinus* and *Striatopodocarpites*) is stepwise, with taeniate forms (e.g., *Lueckisporites, Lunatisporites noviaulensis* and *Protohaploxypinus microcorpus*) and simple and cavate spores (e.g., *Densoisporites*) increasing in dominance. Similar patterns showing the stepwise disappearance of characteristic late Permian pollen taxa have been described from other regions in Gondwana, including the Prince Charles and central Transantarctic Mountains in Antarctica[(41–43)]. In both continents, the palynological transition also heralds the last occurrences of the characteristic *Glossopteris* megafloras[(41, 44)].

Because several of the Australian indicator taxa appear in a different order in the Karoo Basin, or are rare or absent[(45, 46)], we use the overall species associations and the relative proportions of major pollen and spore groups to correlate to the Australian biozones. The palynological assemblage from the 29.9 m horizon is not very productive, contains some pollen that were produced by glossopterids (*Protohaploxypinus*), and is dominated by algal remains simple spores. It is not the typical late Permian assemblage that we expect from a non-disturbed glossopterid woodland. This assemblage might correspond to a transitional stage during the floral turnover, and our best guess is that it falls within the *Playfordia crenulata*. The palynological assemblage at 40.42 m is also dominated by algal remains, such as *Leiosphaeridia* sp., but the terrestrial components are quite different. Based on the absence of pollen produced by glossopterids (e.g., *Protohaploxypinus* and *Striatopodocarpites*), the presence of pollen taxa that become dominant in latest Permian and Triassic palynofloras (*Falcisporites* and

Lunatisporites), the relatively high abundance and diversity of simple spores, and the presence of the cavate spore *Densoisporites*, we consider this younger Nooitgedacht assemblage contemporaneous and equivalent to the *Protohaploxypinus microcorpus* Zone(39). A recent paper by Fielding et al.(38) shows that the *Playfordia crenulata* Zone (palynologically considered equivalent to our 29.9 m assemblage) starts at 252.31 ± 0.07, an age that is indistinguishable from our age determination for the Nooitgedacht ash bed. This would mean that the collapse in *Glossopteris* dominance observed in the Karoo Basin (29.9 m assemblage) is not time-equivalent, but older than end-Permian marine extinction (between 251.941 ± 0.037 and 251.880 ± 0.031 Ma)(47) and, with the data now available, that the floral turnover is contemporaneous across the Gondwanan realm.

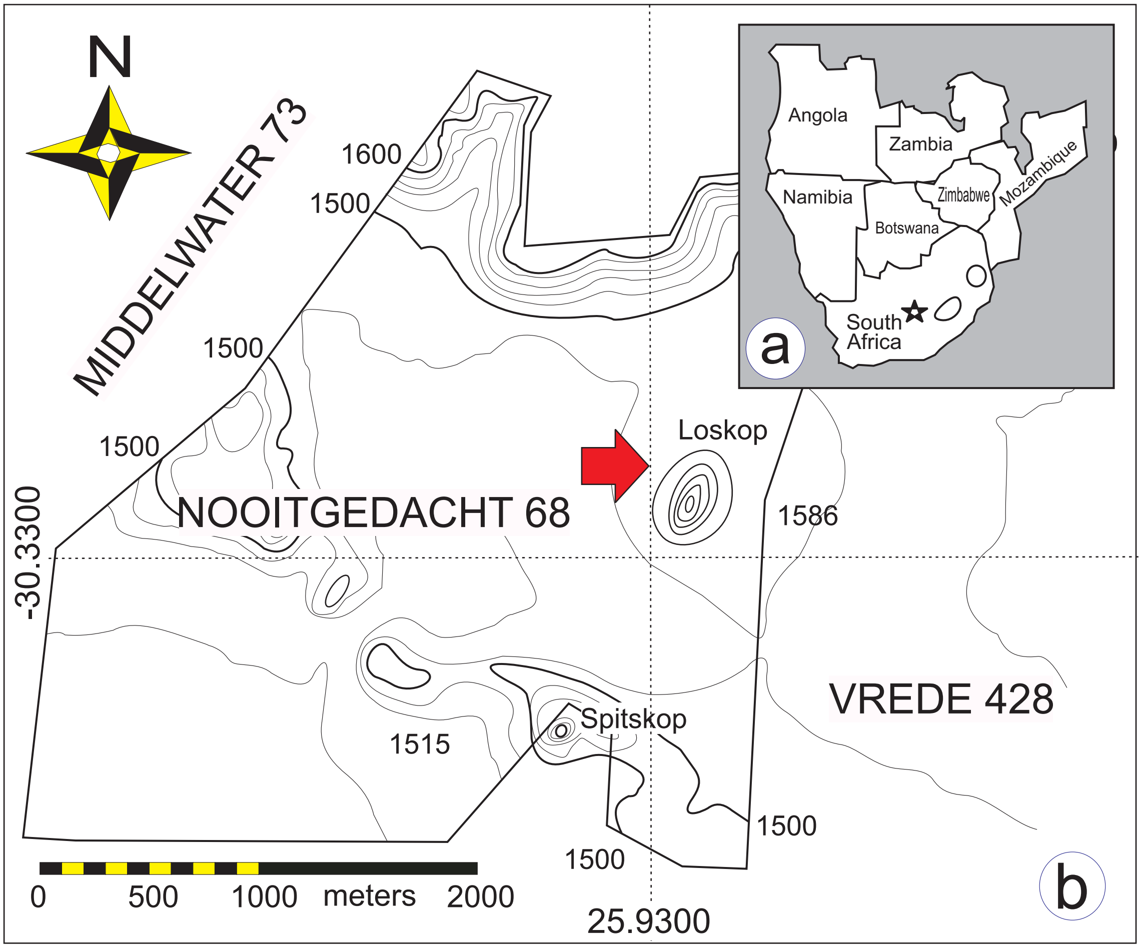

Supplementary Figure 1. Locality maps. (a) Generalized location of farm Nooitgedacht in South Africa. (b) Topographic map on which two koppies (hills), Loskop and Spitskop, are identified from which vertebrates have been reported. Red arrow marks the site of the current study.

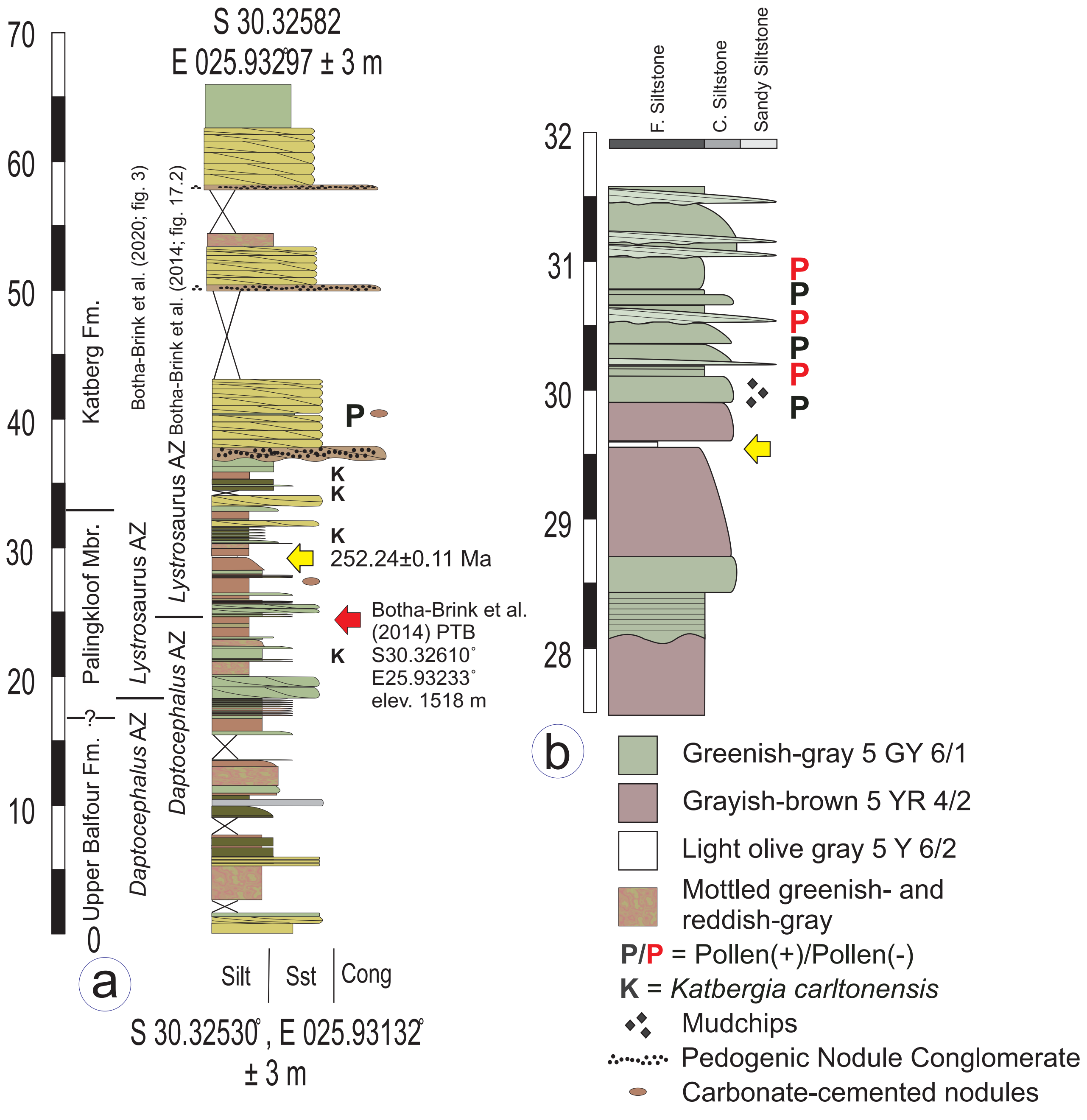

Supplementary Figure 2. Measured stratigraphic section (a) with expanded section between 27 and 32 m showing the interval in which the air-fall ash is located (b). Palynological samples in black yielded pollen and spores; sample horizons in red were barren or low content.

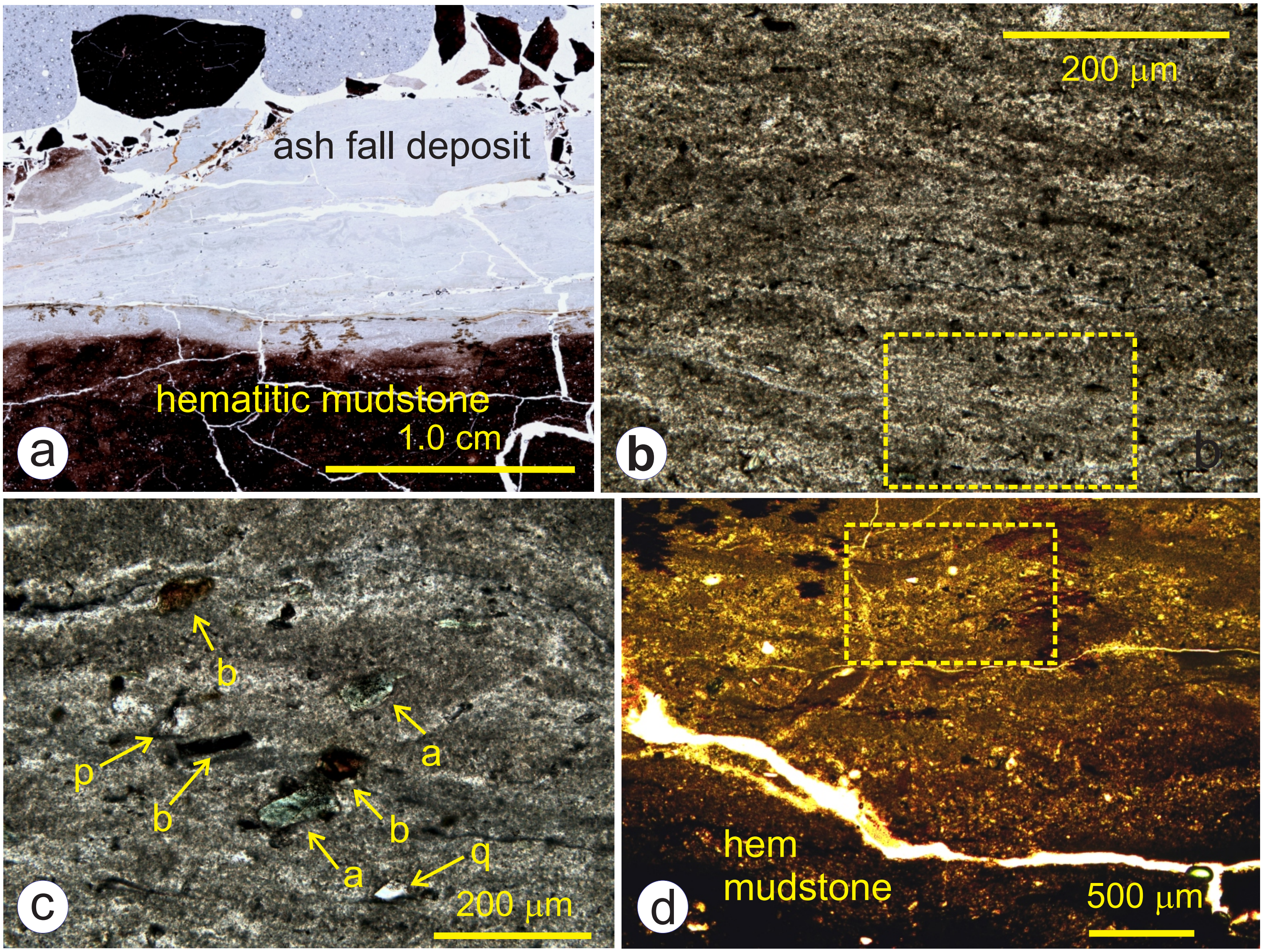

Supplementary Figure 3. Petrographic images of the ash fall deposit and host hematitic mudstone sampled at site 2950–2963 m in the Nooitgedacht section for high precision U-Pb zircon geochronology. (a) Scan of most of the large format uncovered thin section prepared from a field-impregnated sample of the ash bed and underlying mudstone. Scanned image (note scale bar) obtained using a GIGAmacro Magnify2 photo stand with an attached Canon Rebel T5i digital camera. (b) Transmitted light (uncrossed polars) photomicrograph of a part of the ash bed (note scale bar); area highlighted by dotted yellow rectangle includes well-expressed cross-stratification features in the deposit. (c) Transmitted light (uncrossed polars) photomicrograph of another part of the ash bed (note scale bar); arrows point to specific mineral fragments in the deposit (q-quartz, a-amphibole, b-biotite, p-plagioclase). (d) Transmitted light (crossed polars) photomicrograph of another part of the ash bed (note scale bar); area highlighted by dotted yellow rectangle includes well-expressed cross-stratification features in the deposit.

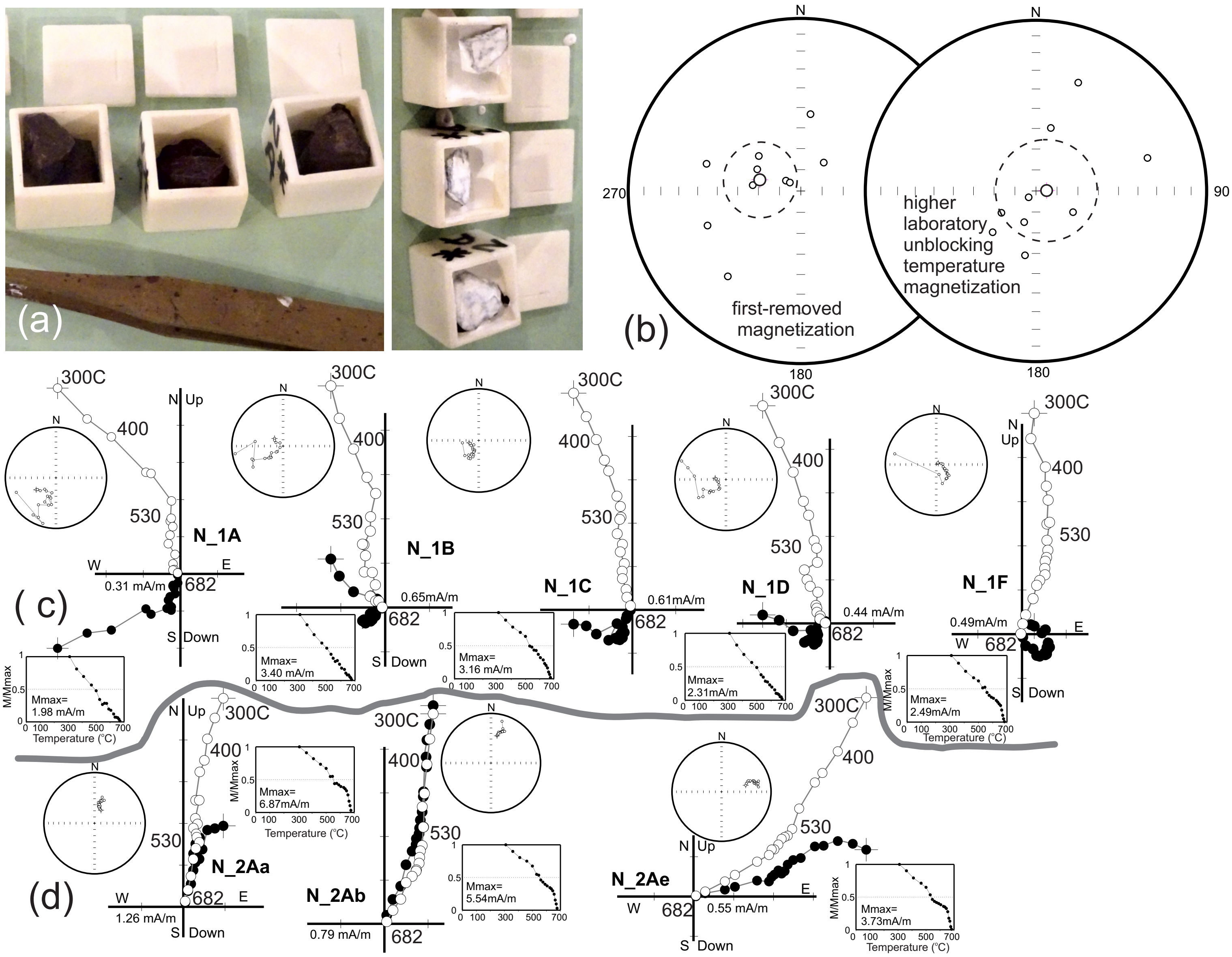

Supplementary Figure 4. P aleomagnetic results from thinly bedded hematitic siltstones/mudstones collected from stratigraphic intervals within five centimeters above and below the ash fall deposit sampled for U-Pb zircon high precision geochronology. (a) Images of oriented (with respect to geographic north and the vertical) hematitic mudstone fragments in ceramic cubes, without and with non-magnetic alumina cement. Ceramic cubes are 20 mm on a side. (b) Equal area projections showing the directions of magnetization (first-removed and higher laboratory unblocking temperature interval) vectors isolated in progressive thermal demagnetization. (c) Examples of progressive thermal demagnetization results from specimens collected immediately above the ash fall deposit. Orthogonal plots showing the endpoint of the magnetization vector plotted on the horizontal (solid circles) and vertical (open circles) planes. Also shown are equal area projections of the remanence vector as a function of progressive demagnetization and normalized (to 300°C) plots of the remanence intensity as a function of progressive thermal demagnetization. (d) same as (c) but for specimens collected immediately below the ash fall deposit.

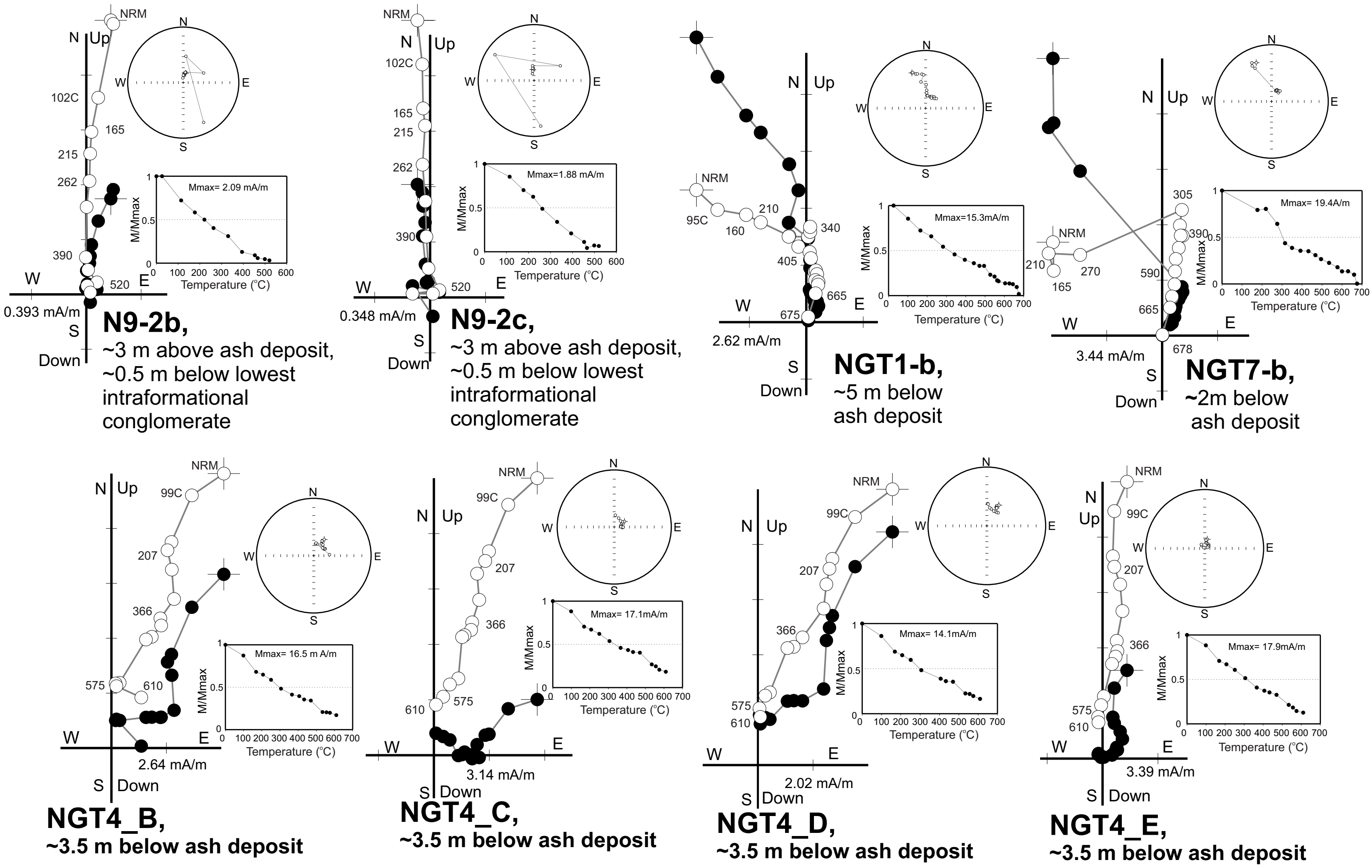

Supplementary Figure 5. Paleomagnetic results from thinly bedded hematitic siltstones/mudstones collected from additional stratigraphic intervals within five meters above and below the ash fall deposit sampled for U-Pb zircon high precision geochronology. Examples of progressive thermal demagnetization results; orthogonal plots showing the endpoint of the magnetization vector plotted on the horizontal (solid circles) and vertical (open circles) planes. Also shown are equal area projections of the remanence vector as a function of progressive demagnetization and normalized (to room temperature) plots of the remanence intensity as a function of progressive thermal demagnetization.

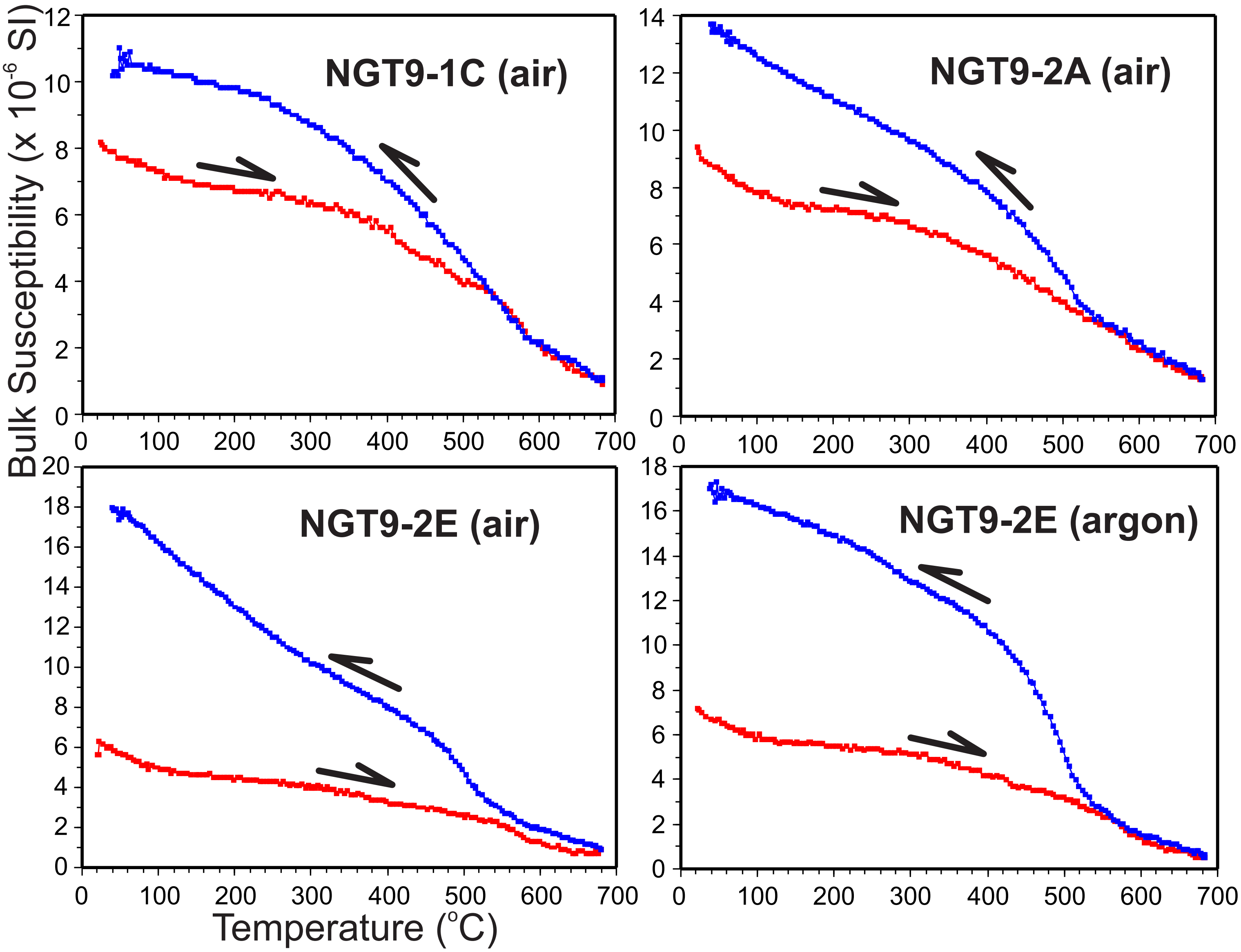

Supplementary Figure 6. Results of continuous bulk susceptibility measurements as a function of heating from room temperature (red curves) to 685°C and cooling back to room temperature (blue curves). Measurements made using an AGICO CS4 thermal apparatus interfaced with an AGICO MFK1-A susceptibility system. Powdered specimens with a mass of about 0.3 gm are used, with most experiments in air and one in argon gas.

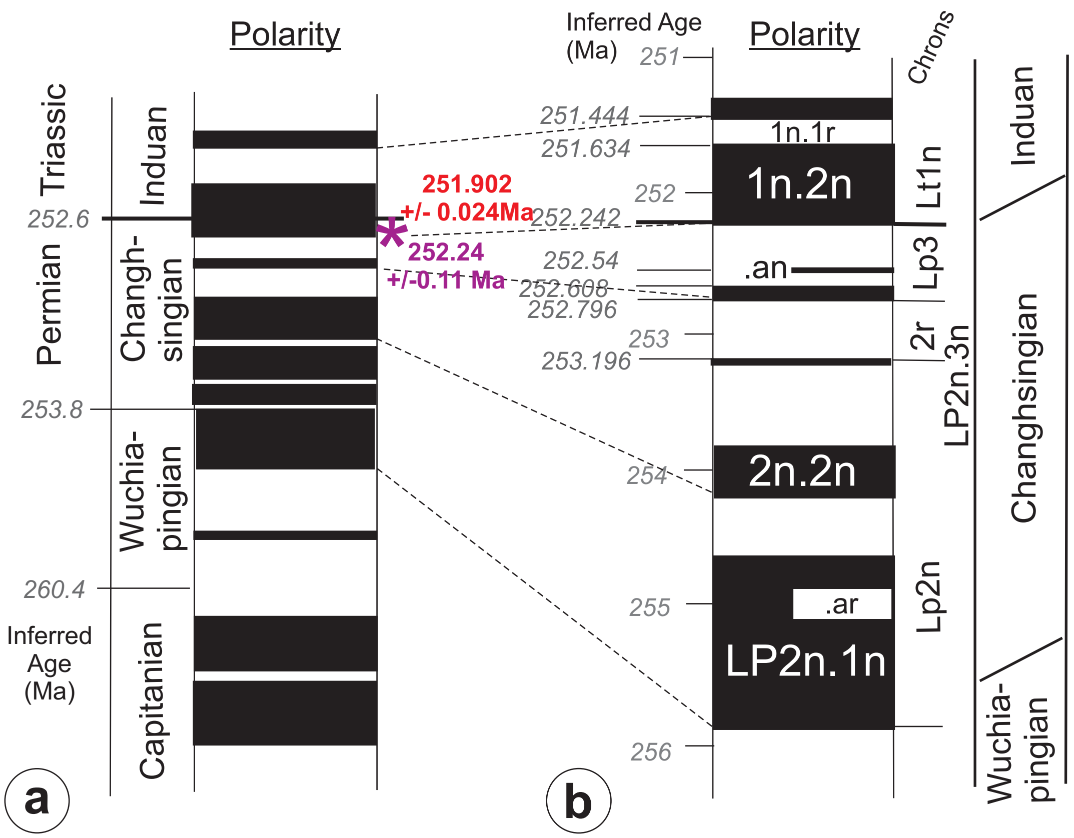

Supplementary Figure 7. E stimates of the geomagnetic polarity time scale from the late Permian to earliest Triassic, modified from (a) Szurlies (2013) and (b) Hounslow and Balabanov (2016). Inferred ages for stage boundaries (a) or for the start and/or end of specific polarity chrons (B) are taken directly from the original publication and have not been modified as a result of more recent revisions to the Geologic Time Scale. Black (white) refers to normal (reverse) polarity chrons. Labelled polarity chrons in (b) are directly from the source. Note that the vertical (inferred age) scales are different for (a) and (b). Dashed lines represent our attempt to correlate the bases of key normal polarity chrons. Note that in (a), the Permian/Triassic boundary is placed within a normal polarity chron, estimated by Szurlies (2013) to be about 700 ka in duration. In (b), the
Permian/Triassic boundary is placed at the base of a normal polarity chron, estimated to be about 600 ka in duration.

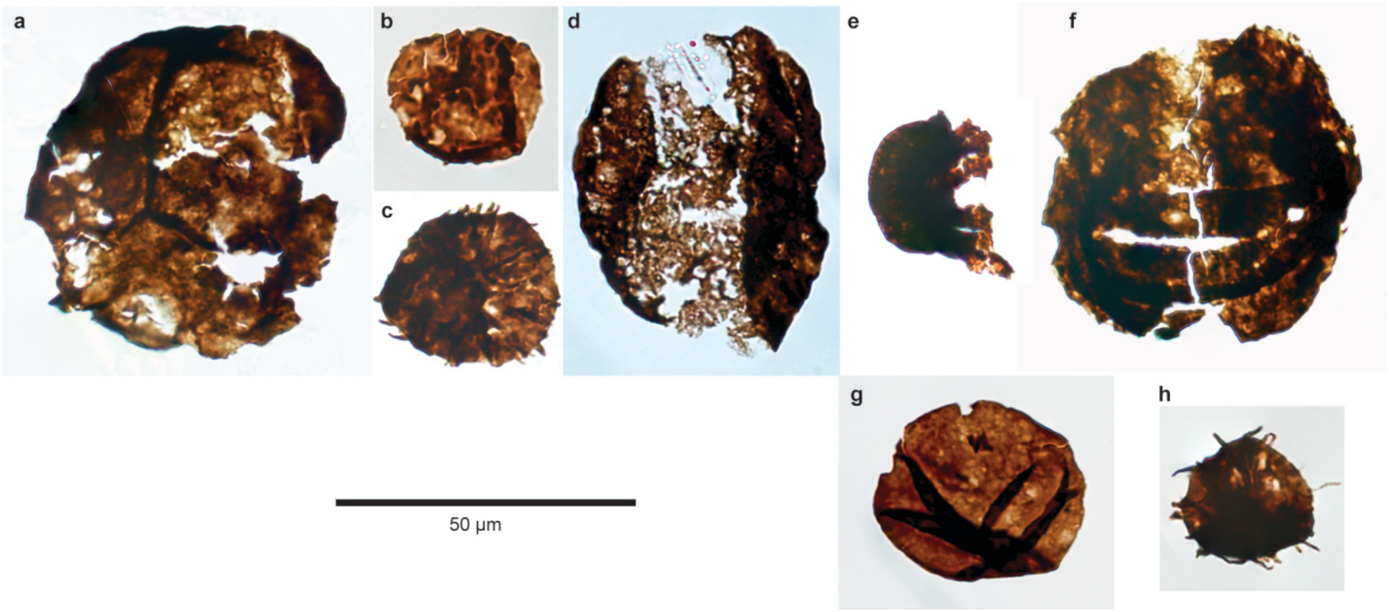

Supplementary Figure 8. Representative palynomorphs recovered from greenish-gray coarse siltstone at a stratigraphic height of 29.9 m (see supplemental Fig. 1; UCMP collection locality number PA1378.02). Scale bar is 50 µm. Taxonomic names are followed by the slide number, England Finder graticule coordinates and the UCMP specimen number.

(a) *Punctatisporites gretensis* Balme and Hennelly 1956. [N2990 P-1, K44, 398665]; **(b)** *Altitriletes* sp. cf. *A. densus* Venkatachala and Kar 1968. [N2990 P-2, Y57-3, 398666]; **(c**) *Brevitriletes cornutus* (Balme & Hennelly) Backhouse 1991. [N2990 P-1, K64-1-2, 398667]; **(d**) Taeniate bisaccate pollen indet. [N2990 P-1, S53-3, 398668]; **(e)** *Lunatisporites* sp. cf. *L. pellucidus* (Goubin) Helby in de Jersey 1972 [N2990 P-1, P45-2, 398669]; **(f**) *Protohaploxypinus* sp. cf. *P. samoilovichii* (Jansonius 1962) Hart 1964 [N2990 P-1, H57-1, 398670]; **(g)** *Leiosphaeridia* sp. [N2990 P-2, M46-1/3, 398671]; **(h)** *Micrhystridium* sp. [N2990 P-2, W41-4, 398672]

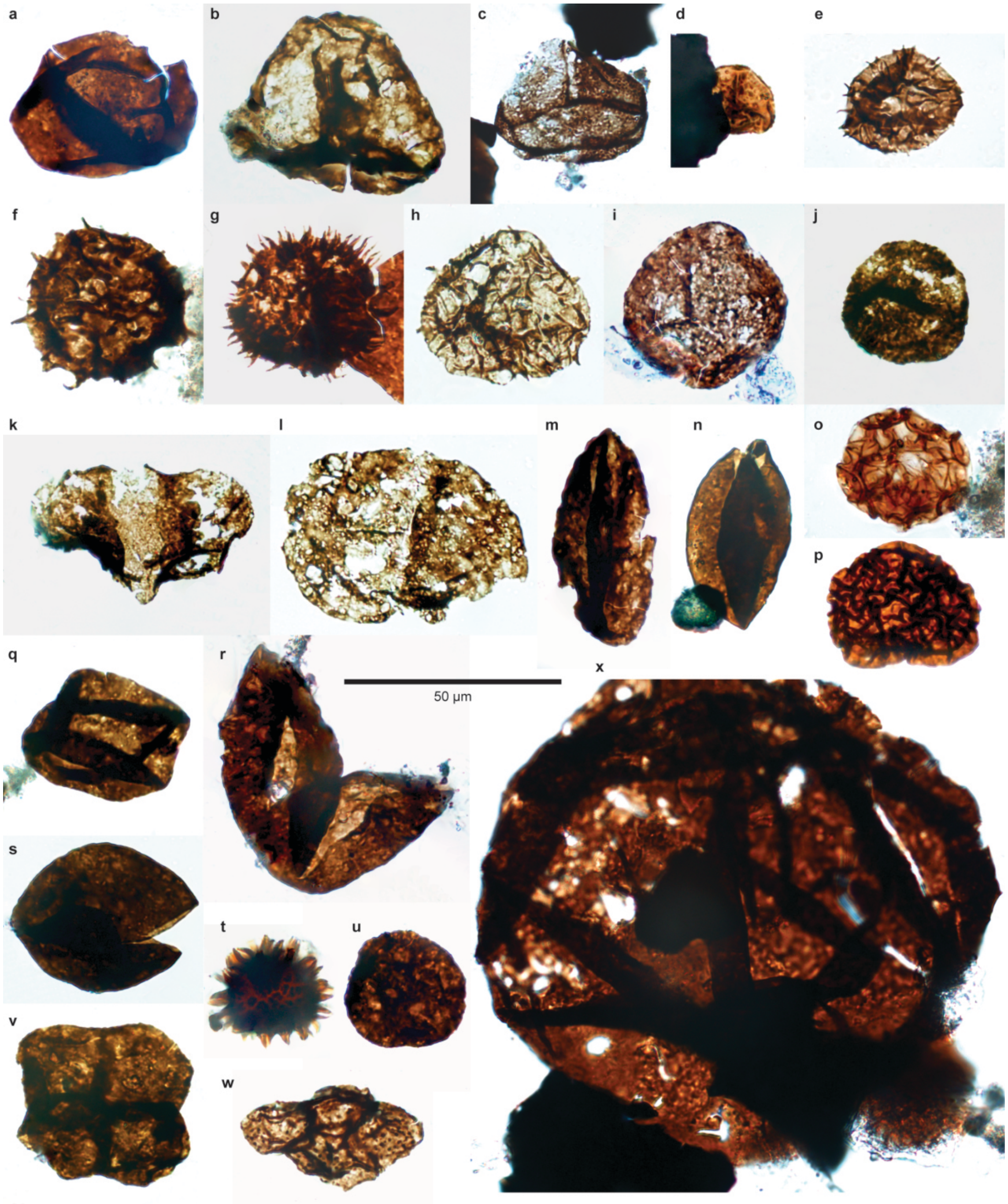

Supplementary Figure 9. Representative palynomorphs recovered from greenish-gray coarse siltstone at a stratigraphic height of 40.42 m (see supplemental Fig. 1; UCMP collection locality number PA1378.01). Scale bar is 50 µm. Taxonomic names are followed by the slide number, England Finder graticule coordinates and the UCMP specimen number.

(**a**) *Punctatisporites priscus* Bharadwaj and Salujha 1965 [N4042 P-1, P38-4, 398673]**;** **(b)** *Leiotriletes directus* Balme and Hennelly 1956 [N4042 P-2, Q47-4, 398674]; (**c**) *Osmundacidites wellmanii* Couper 1953 [N4042 P-1, X59-1-2, 398675]; (**d**) *Lophotriletes* sp. cf. *L. novicus* Singh 1964 [N4042 P-1, U60-2, 398676]; **(e)** *Micrhystridium* sp. [N4042 P-1, E53-1, 398677]; **(f)** *Brevitriletes cornutus* (Balme and Hennelly) Backhouse 1991 [N4042 P-1, R54-1, 398678]; **(g)** *Horriditriletes filiformis* (Balme and Hennelly) Backhouse 1991 [N4042 P-2, E59-4, 398679]; **(h)** *Horriditriletes* sp. cf. *H. tereteangulatus* (Balme and Hennelly) Backhouse 1991 [N4042 P-2, S55-1, 398680] ; **(i)** *Limatulasporites* sp. (Balme) Helby and Foster 1979 in Foster, 1979 [N4042 P-1, D55-1, 398681]; **(j)** *Densoisporites nejburgii* (Schulz 1964) Balme 1970 [N4042 P-2, K43-1, 398682]; (**k**) *Falcisporites australis* (de Jersey) Stevens 1981 [N4042 P-2, R43-3, 398683]; (**l**) cf. *Lueckisporites virrkiae* Potonié and Klaus 1954 [N4042 P-2, R43-3, 398684]; (**m**) cf. *Ephedripites* sp. (Bolkhovitina) Potonié 1985 [N4042 P-1, T51-3, 398685]; (**n**) *Cycadopites cymbatus* (Balme and Hennelly) Segroves 1970 [N4042 P-2, E51-1, 398686]; (**o**) *Reticulatisporites pseudopalliatus* Staplin 1960 [N4042 P-1, S60-4, 398687]; (**p**) *Rugaletes playfordii* Foster 1979 [N4042 P-1, P36-3, 398688]; (**q**) *Leiosphaeridia* sp. Eisenack 1958 [N4042 P-1, F47-1_3, 398689]; (**r-s**) *Brazilea* sp. Tiwari and Navale 1967 [N4042 P-1, K38-3, 398690] [N4042 P-2, S55-1, 398691]; (**t**) *Mehlisphaeridium fibratum* Segroves 1967 [N4042 P-2, L46, 398692]; (**u**) *Leiosphaeridia* sp. [N4042 P-1, K43-1, 398693]; (**v-w** - *Quadrisporites horridus* Hennely 1958 [N4042 P-1, O52-3, 398694] [N4042 P-2, L46, 398695]; (**x**) Large (mega)spore indet. [N4042 P-1, F52-2, 398696]

Supplementary Table 1. U-Pb isotopic data for single, chemically abraded zircon grains from an ash bed on the Farm Nooitgedacht 68, Bethulie District, Free State Province, South Africa.

No.	Weight (μg)	a) Pb_C (pg)	b) Pb_T/ Pb_C	U (ppm)	c) Th/U	d) $^{206}Pb/^{204}Pb$	e) $^{207}Pb/^{235}U$	2σ	e) $^{206}Pb/^{**238}U$	2σ	f) Err Corr	$^{207}Pb/^{**206}Pb$	2σ	Age (Ma) $^{207}Pb/^{**206}Pb$	2σ	Age (Ma) $^{207}Pb/^{235}U$	2σ	Age (Ma) $^{206}Pb/^{*238}U$	2σ	% Disc
ash bed, Farm Nooitgedacht 68 (S30' 32616º, E 025' 93242º, 1525 m), Bethulie District, Free State Province																				
z1*	1.0	0.16	17.0	66	0.46	1079	0.2834	0.0051	0.040222	0.000110	0.526	0.05110	0.00085	245	38	253.4	4.0	254.21	0.68	-3.6
z2*	2.3	0.47	7.1	31	0.94	411	0.2822	0.0044	0.039946	0.000114	0.531	0.05124	0.00072	252	32	252.4	3.4	252.50	0.71	-0.3
z3*	1.6	0.18	23.8	52	1.45	1194	0.2793	0.0034	0.039946	0.000097	0.472	0.05070	0.00057	227	26	250.1	2.7	252.50	0.60	-11
z4*	1.0	0.17	23.9	79	1.57	1168	0.2843	0.0026	0.039930	0.000096	0.500	0.05164	0.00042	269	18	254.1	2.0	252.40	0.59	6.5
z5	1.1	0.23	21.0	84	1.50	1049	0.2828	0.0036	0.039929	0.000055	0.464	0.05137	0.00062	257	28	252.9	2.8	252.40	0.34	1.9
z6	2.2	0.76	5.5	36	1.55	283	0.2828	0.0098	0.039928	0.000058	0.462	0.05138	0.00174	258	78	252.9	7.7	252.39	0.36	2.1
z7	2.1	1.8	4.9	81	1.35	267	0.2827	0.0102	0.039927	0.000063	0.406	0.05136	0.00182	257	82	252.8	8.1	252.38	0.39	1.9
z8	1.3	1.6	2.3	52	1.62	129	0.2849	0.0222	0.039903	0.000110	0.471	0.05178	0.00397	276	179	254.5	17.6	252.23	0.68	8.7
z9	1.9	0.30	44.7	137	1.48	2216	0.2830	0.0016	0.039900	0.000050	0.405	0.05144	0.00028	261	12	253.1	1.3	252.22	0.31	3.4
z10	1.5	0.26	34.4	108	1.67	1649	0.2833	0.0022	0.039901	0.000053	0.390	0.05149	0.00038	263	17	253.3	1.8	252.22	0.33	4.2
z11	1.8	0.27	18.5	53	1.53	919	0.2835	0.0037	0.039891	0.000042	0.488	0.05155	0.00064	265	29	253.5	2.9	252.16	0.26	5.1
z12	1.3	1.5	5.0	111	1.59	257	0.2805	0.0103	0.039889	0.000073	0.374	0.05100	0.00185	241	84	251.1	8.2	252.15	0.45	-4.8
z13	1.4	0.32	31.9	103	1.42	1187	0.2838	0.0027	0.039873	0.000048	0.398	0.05162	0.00047	268	21	253.6	2.1	252.04	0.30	6.2
z14*	1.0	0.16	29.1	78	2.31	1233	0.2849	0.0026	0.039855	0.000094	0.496	0.05185	0.00042	279	19	254.6	2.0	251.93	0.58	9.9

Notes:

Zircon grains were chemically abraded ('CA', Mattinson, 2005). Errors are 2σ absolute.

Asterisks (z1-4, z14) denote analyses using in-house 'ROM' spike, all others used ET535 from the EARTHTIME Project.

a) total common Pb in picograms; assumed isotopic composition of laboratory blank ($^{206}Pb/^{204}Pb$=18.49±0.4%; $^{207}Pb/^{204}Pb$=15.59±0.4%;$^{208}Pb/^{204}Pb$=39.36±0.4%).

b) ratio of total Pb in the analysis (radiogenic and common) to total common Pb

c) Th/U calculated from radiogenic $^{208}Pb/^{206}Pb$ ratio and $^{207}Pb/^{206}Pb$ age assuming concordance.

d) $^{206}Pb/^{204}Pb$ corrected for fractionation and common Pb in the spike.

e) Pb/U ratios corrected for fractionation, common Pb in the spike, and blank.

f) Error Corr is correlation coefficients of X-Y errors on the concordia plot.

**Correction for ^{230}Th disequilibrium in $^{206}Pb/^{238}U$ and $^{207}Pb/^{206}Pb$ assuming Th/U of 4.2 in the magma.

Decay constants are those of Jaffey et al. (1971): ^{238}U and ^{235}U are 1.55125 x 10^{-10}/yr and 9.8484 x 10^{-10}/yr.

$^{238}U/^{235}U$ ratio of 137.88 used for $^{207}Pb/^{206}Pb$ model age calculation.

Supplementary Table 2. Paleomagnetic results from hematitic siltstone samples collected, as packed ceramic cubes, at different stratigraphic levels below and above an ash bed on the Farm Nooitgedacht 68, Bethulie District, Free State Province, South Africa.

	a)	b)	c)	d)	e)	f)	c)	d)
Sample	C1, Decl.	C1, Incl.	Demag. Interval	MAD	C2, Decl.	C2, Incl.	Demag. Interval	MAD
Horizon N9_2, about 3.0 m above ash deposit								
N9_2a					229.4	-73.4	221-466	16.0
N9_2b					160.4	-81.5	177-499	7.7
N9_2c					314.9	-84.4	221-453	5.1
N9_2d					333.1	-73.3	221-453	3.3
N9_2e								
Horizon N1, within 5.0 cm above ash deposit								
N1_A	249.7	-41.9	310-525	3.5	189.7	-58.6	545-655	12.7
N1_B	309.8	-64.1	310-460	5.8	229.2	-85.4	500-645	13.7
N1_C	276.9	-67.1	310-500	9.0	200.7	-74.1	545-663	7.8
N1_D	296.5	-67.0	310-530	5.3	226.4	-61.3	575-663	10.2
N1_E	304.8	-81.5	310-460	3.5	237.6	-70.5	545-663	10.3
N1_F	307.9	-83.5	310-460	6.2	119.8	-69.6	560-676	9.1
Horizon N1, within 5.0 cm below ash deposit								
N2_Aa	38.9	-72.8	310-460	5.5	13.3	-58.9	545-663	8.6
N2_Ab	7.2	-53.0	310-530	3.3	21.3	-32.4	605-676	2.2
N2_Ac	220.5	-35.2	310-460	2.9	222.7	-13.3	605-676	4.5
N2_Ad	286.1	-42.6	310-545	6.4	356.6	-4.9	605-670	6.4
N2_Ae	260.4	56.7	310-460	6.7	73.5	-32.4	560-676	4.9
Horizon NGT9, about 1.3 m below ash deposit								
NGT9_A	341.7	-6.0	192-252	5.3	168.0	-25.4	252-672	19.6
NGT9_B	290.1	-25.3	105-252	11.6	110.8	-48.5	531-672	11.4
NGT9_C	29.0	-26.6	105-230	2.4	203.9	-42.4	531-672	3.7
NGT9_D	313.2	-29.2	105-230	1.7	126.1	-44.5	577-672	6.4
NGT9_E	354.9	-33.7	105-230	0.7	174.8	-54.2	531-672	7.2
Horizon NGT8, about 1.6 m below ash deposit								

NGT8_A	107.0	-60.3	162-292	6.5				
NGT8_B	179.4	-55.9	162-292	4.7				
NGT8_C	155.3	-64.1	162-292	3.3				
NGT8_D	177.9	-42.2	162-292	4.2	179.1	-19.4	520-667	14.0
<u>Horizon NGT7, about 2 m below ash deposit</u>								
NGT7_A	331.3	-7.5	177-315	6.0	134.8	-64.7	495-675	13.4
NGT7_B	301.2	-21.8	219-315	5.7	123.6	-71.2	350-660	4.5
NGT7_C	357.9	-23.3	177-392	8.5	135.7	-61.4	535-682	16.1
NGT7_D	329.1	-52.6	219-392	8.4	30.3	-78.0	432-630	8.3
NGT7_E	22.3	-6.4	177-315	5.4	211.8	-66.4	432-660	6.7
<u>Horizon NGT5, about 3 m below ash deposit</u>								
NGT5_A					344.1	-69.4	345-684	11.8
NGT5_B					267.9	-67.3	345-604	8.3
NGT5_C	118.3	-67.5	103-292	9.4	22.2	-83.0	345-657	8.9
NGT5_D	145.9	-43.6	103-251	11.4	192.0	-71.1	345-667	13.1
NGT5_E	103.0	-49.8	103-292	7.1	257.0	-69.4	381-635	10.6
<u>Horizon NGT4, about 3.5 m below ash deposit</u>								
NGT4_A	332.1	-52.3	169-386	1.8	99.8	-79.4	470-575	3.2
NGT4_B					82.3	-55.3	306-556	3.9
NGT4_C	33.4	-58.5	99-306	5.9	128.2	-65.4	433-610	11.9
NGT4_D	14.7	-36.6	99-306	6.3	61.0	-59.1	406-610	6.3
NGT4_E	350.4	-62.1	99-306	5.5	82.0	-74.9	433-610	4.7
<u>Horizon NGT2, about 4.5 m below ash deposit</u>								
NGT2_C					82.7	-70.7	392-675	12.7
NGT2_D					156.5	-67.6	315-675	18.4
NGT2_E					146.5	-54.8	467-675	9.7
NGT2_F					125.0	-80.6	315-682	16.3
<u>Horizon NGT1, about 5 m below ash deposit</u>								
NGT1_A					35.7	-79.4	165-626	13.7
NGT1_B					330.1	-48.8	221-570	18.7
NGT1_C	56.6	-40.6	165-395	10.4	341.9	-59.7	471-675	14.0
NGT1_D	357.4	-50.1	165-442	16.9	126.7	-50.1	563-675	7.4
NGT1_E	3.5	-31.9	165-442	16.5	349.5	-78.9	342-675	22.1
NGT1_F	334.9	-48.0	221-563	13.9	144.1	-39.7	563-675	30.5

NGT1_G	349.3	-54.6	165-442	13.3	174.6	-85.3	563-675	12.2

Notes:

All data expressed in geographic coordinates.

a) declination, in degrees east of north, of the first-removed component of remanence (if any).
b) inclination, in degrees positive downwards and negative upwards, of the first-removed component of remanence (if any).
c) Laboratory unblocking temperature interval (in ^{o}C) over which first- or second- removed component is unblocked.
d) Maximum angular deviation (in ^{o}) result for first- or second-removed remanence component; always determined on the basis of four or usually several more data points, using free-floating (completely unanchored) lines, and not the origin. Alternate approaches would likely result in lower MAD values.
e) Declination, in degrees east of north, of the second-removed component of remanence.
f) Inclination, in degrees positive downwards and negative upwards, of the second-removed component of remanence.

Supplementary Table 3. Analytical results for TOC and Hg (ppb) from two short stratigraphic intervals on Nooitgedacht farm, and a lot of Hg/%C versus %C.

	Hg (ppb)	%C (EA)	Stratigraphic Position	Hg/%C
NOOITGEDACHT Sample				
210319.4	0.99	0.07	30 cm above ash in GG siltstone	14.14
210319.1	1.07	0.08	5 cm above ash in RG siltstone	13.38
210319.2	3.62	0.31	0 cm = ash	11.68
210319.3	0.73	0.07	-5 cm below ash in RG siltstone	10.43
210319.5	0.84	0.04	30 cm above "PTB" in RG siltstone	21.00
210319.6	0.53	0.05	10 cm above "PTB" in RG siltstone	10.60
210319.7	1.93	2.3	Nodule above "PTB"	0.84
210319.7 IR	0.31	0.06		5.17
210319.8	0.83	0.21	-5 cm below "PTB" in GG siltstone	3.95
210319.9	0.65	0.07	-20 cm below 'PTB' at contact GG/RG si	9.29
210319.10	0.59	0.03	-30 cm below "PTB" in RG siltstone	19.67
IR = Insoluble Residue				
GG = Greenish Gray				
RG = Reddish/Brownish Gray				

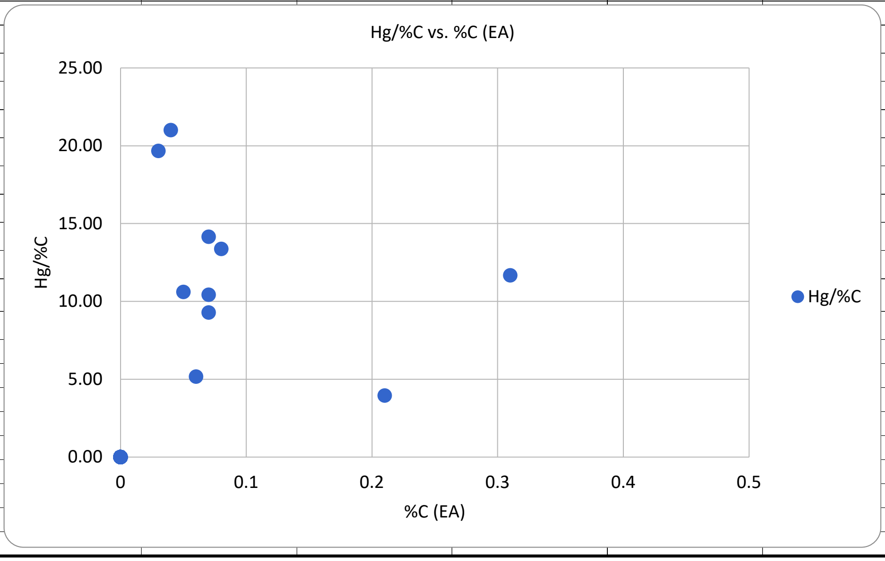

Supplementary References

1. Gastaldo R.A., Neveling,J., Clark, C.K. and Newbury, S.S. The terrestrial Permian-Triassic boundary event bed is a non-event. *Geology,* 37, 199 – 202 (2009).
2. Gastaldo, R.A., Neveling, J., Geissman, J.W. & Li, J. A multidisciplinary approach to review the vertical and lateral facies relationships of the purported vertebrate-defined terrestrial Permian–Triassic boundary interval at Bethulie, Karoo Basin, South Africa. *Earth-Science Reviews*, **189**, 220-243 (2019).
3. Smith, R. M. H. & Botha-Brink, J. Anatomy of a mass extinction: Sedimentological and taphonomic evidence for drought-induced die-offs at the Permo–Triassic boundary in the main Karoo Basin, South Africa: *Palaeogeogr. Palaeoclimatol. Palaeoecol.*, **396**, 99–118 (2014).
4. Botha, J., Huttenlocker, A. K., Smith, R. M. H., Prevec, R., Viglietti, P., & Modesto, S. P. New geochemical and palaeontological data from the Permian-Triassic boundary in the South African Karoo Basin test the synchronicity of terrestrial and marine extinctions. *Palaeo Palaeo Palaeo* 540, 109467 (2020).
5. Botha-Brink, J., Huttenlocker, A. K., & Modesto, S. P. Vertebrate Paleontology of Nooitgedacht 68. A *Lystrosaurus maccaigi*-rich Permo-Triassic Boundary Locality in South Africa. *in* Kammerer, C.F., Angielczyk, K.D., & Fröbisch, J., eds., Early Evolutionary History of the Synapsida, Vertebrate Paleobiology and Paleoanthropology. Springer, Dordrecht, 289–304 (2014). doi. 10.1007/978-94-007-6841-3_17
6. Pace, D. W., Gastaldo, R. A., & Neveling, J. Early Triassic aggradational and degradational landscapes of the Karoo basin and evidence for climate oscillation following the P-Tr Event. *J. Sed. Res.* **79**, 316–331 (2009).

7. Gastaldo, R. A., Kamo, S. L., Neveling, J., Geissman, J. W., Bamford, M. & Looy, C. V. Is the vertebrate defined Permian–Triassic Boundary in the Karoo Basin, South Africa, the terrestrial expression of the End Permian marine event? *Geology* **43**, 939–942 (2015). doi.10.1130/G37040.1.

8. Gastaldo, R. A., Neveling, J., Looy, C. V., Bamford, M. K., Kamo, S. L., & Geissman, J.W. Paleontology of the Blaauwater 67 Farm, South Africa: Testing the *Daptocephalus/Lystrosaurus* Biozone Boundary in a Stratigraphic Framework. *PALAIOS* **34**, 349–366 (2017).

9. Gastaldo, R. A., Neveling, J., Geissman, J. W., & Kamo, S. L. A Lithostratigraphic and Magnetostratigraphic Framework in a Geochronologic Context for a Purported Permian–Triassic Boundary Section at Old (West) Lootsberg Pass, Karoo Basin, South Africa. *Geol. Soc. Am. Bull.* **130**, 1411–1438 (2018). https.// doi .org /10 .1130 /B31881 .1

10. Gastaldo, R. A., Neveling, J., Geissman, J. W., & Looy, C. V. Testing the *Daptocephalus* and *Lystrosaurus* Assemblage Zones in a Lithostratographic, Magnetostratigraphic, and Palynological Framework in the Free State, South Africa. *PALAIOS* **34**, 542– 561 (2019). DOI: http://dx.doi.org/10.2110/palo.2019.019.

11. Szurlies, M. Late Permian (Zechstein) magnetostratigraphy in Western and Central Europe: *Geol. Soc. London Spec. Pub.* **376**, (2013) doi:10.1144/SP376.7.

12. Hounslow, M. W., & Balabanov, Y. P. A geomagnetic polarity time scale for the Permian, calibrated to stage boundaries. *Geol. Soc. London Spec. Pub.* **450**, (2016) https.//doi.org/10.1144/SP450.8.

13. Font, E., Adatte, T., Sial, A. N., de Lacerda, L. D., Keller, G., & Punekar, J. Mercury anomaly, Deccan volcanism, and the End-Cretaceous mass extinction. *Geology* **44,** 171–174 (2016).

14. Thibodeau, A. M., Ritterbush, K., Yager, J. A., West, A. J., Ibarra, Y., Bottjer, D. J., Berelson, W. A., Bergquist, B. A., & Corsetti, F. A. Mercury anomalies and the timing of biotic recovery following the End-Triassic mass extinction. *Nature Commun*. **7**, 11147 (2016).

15. Jones, D. S., Martini, A. M., Fike, D. A., & Kaiho, K. A volcanic trigger for the Late Ordovician mass extinction? Mercury data from South China and Laurentia. *Geology* **45,** 631–634 (2017).

16. Grasby, S. E., Shen, W., Yin, R., Gleason, J. D., Blum, J. D., Lepak, R. F., Hurley, J. P., & Beauchamp, B. Isotopic signatures of mercury contamination in latest Permian oceans. *Geology* **45,** 55–58 (2017).

17. Racki, G., Rakociñski, M., Marynowski, L., & Wignall, P.B. Mercury Enrichments and the Frasnian-Famennian Biotic Crisis: A Volcanic Trigger Proved? *Geology* **46,** 543–546 (2018).

18. Wang, X., Cawood, P. A., Zhao, H., Zhao, L., Grasby, S. E., Chen, Z.-Q., & Zhang, L. Global Mercury Cycle during the End-Permian Mass Extinction and Subsequent Early Triassic Recovery. *Earth Planet Sci Lett* **513**, 144–155 (2019).

18. Shen, J., Chen, J., Algeo, T. J., Yuan, S., Feng, Q., Yu, J., Zhou, L, O'Connell, B., & Planavsky, N. J. Evidence for a prolonged Permian-Triassic extinction interval from global marine mercury records. *Nature Commun.* **10**, 1563 (2019).

20. Percival, L. M. E., Ruhl, M., Hesselbo, S. P., Jenkyns, H. C., Mather, T. A. & Whiteside, J. H. Mercury evidence for pulsed volcanism during the end-Triassic mass extinction. *Proc. Nat. Acad. Sci. USA* **114**, p. 7929–7934 (2017).

21. Chu, D., Grasby, S. E., Song, H., Dal Corso, J., Wang, Y., Mather, T. A., Wu, Y., Song, H., Shu, W., Tong, J., & Wignall, P. B. Ecological disturbance in tropical peatlands prior to marine Permian-Triassic mass extinction. *Geology* **48**, https://doi.org/10.1130/G46631.1

22. Balme, B. E. Fossil *in situ* spores and pollen grains: an annotated catalogue. *Rev. Palaeobot. Palynol.* **87**, 81–323 (1995).

23. Grauvogel-Stamm, L., & Lugardon, B. The spores of the Triassic lycopsid *Pleuromeia sternbergii* (Munster) Corda: morphology, ultrastructure, phylogenetic implications, and chronostratigraphic inferences. *Int. J. Plant Sci.* **165**, 631–650 (2004).

24. Looy, C. V., Collinson, M. E., Van Konijnenburg-van Cittert, J. H. A., & Visscher, H. The ultrastructure and botanical affinity of end-Permian spore tetrads. *Int. J. Plant Sci.* **166**, 875–887 (2005).

25. Zavada, M. S. The ultrastructure of pollen found in dispersed sporangia of *Arberiella* (Glossopteridaceae). *Bot. Gaz.* **152**, 248–255 (1991).

26. Lindström, S., McLoughlin, S., & Drinnan, A. N. Intraspecific variation of taeniate bisaccate pollen within Permian glossopterid sporangia, from the Prince Charles Mountains, Antarctica. *Int. J. Plant Sci.* **158**, 673–684 (1997).

27. Ryberg, P., Taylor, E. L., & Taylor, T. N. The first permineralized microsporophyll of the Glossopteridales: *Eretmonia macloughlinii* sp. nov. *Int. J. Plant Sci.* **173**, 812–822 (2012).

28. Clement-Westerhof, J. A. *In situ* pollen from gymnospermous cones from the Upper Permian of the Italian Alps - a preliminary account. *Rev. Palaeobot. Palynol.* **17**, 63–73 (1974).

29. Clement-Westerhof, J. A. Aspects of Permian palaeobotany and palynology, VII, the Majonicaceae, a new family of Late Permian conifers. *Rev. Palaeobot. Palynol.* **52**, 375–402 (1987).

30. Retallack, G. J. *Lepidopteris callipteroides*, an earliest Triassic seed fern of the Sydney Basin, southeastern Australia. *Alcheringa* **26**, 475–500 (2002).

31. Zavada, M. S., & Crepet, W. L. Pollen wall ultrastructure of the type material of *Pteruchus africanus*, *P. dubius* and *P. papillatus*. *Pollen Spores* **27**, 271–276 (1985).

32. Taylor, E. L., Taylor, T. N., Kerp, H., & Hermsen, E. J. Mesozoic seed ferns: old paradigms, new discoveries. *J. Torrey Bot. Soc.* **133**, 62–82 (2006).

33. Blomenkemper, P., Kerp, H., Abu Hamad, A., DiMichele, W. A., & Bomfleur, B. A hidden cradle of plant evolution in Permian tropical lowlands. *Science* **362**, 1414–1416 (2018).

34. Abu Hamad, A., Kerp, H., Vörding, B., & Bandel, K. A. Late Permian flora with *Dicroidium* from the Dead Sea region, Jordan. *Rev. Palaeobot. Palynol.* **149**, 85–130 (2008).

35. Chandra, S., Singh, K. J., & Jha, N. First report of the fertile plant genus *Umkomasia* from Late Permian beds in India and its biostratigraphic significance. *Palaeontology* **51**, 817–826 (2008).

36. Metcalfe, I., Crowley, J. L., Nicoll, R. S., & Schmitz, M. High-precision U-Pb CATIMS calibration of middle Permian to lower Triassic sequences, mass extinction and extreme climate-change in eastern Australian Gondwana. *Gond. Res.* **28**, 61–81 (2015).

37. Laurie, J. R., Bodorkos, S., Nicoll, R. S., Crowley, J., Mantle, J. D., Mory, A. J., Wood, G. R., Backhouse, J., Holmes, E. K., Smith, T. E., & Champion, D. C. Calibrating the middle and late Permian palynostratigraphy of Australia to the geologic timescale via U-Pb zircon CA-IDTIMS dating. *Aust. J. Earth Sci.* **63**, 701–730 (2016).

38. Fielding, C. R., Frank, T. D., McLoughlin, S., Vajda, V., Mays, C., Tevyaw, A. P., Winguth, A.,Winguth, C., Nicoll, R. S., Bocking, M., & Crowley, J. L., Age and pattern of the southern high-latitude continental end-Permian extinction constrained by multiproxy analysis. *Nature Comm.* **10**, 385, (2019) doi: 10.1038/s41467-018-07934

39. Mory, A. J., & Backhouse, J. Permian stratigraphy and palynology of the Carnarvon Basin, Western Australia. *Geol. Surv. W. Aust. Rep.* **51**, 1–101 (1997).

40. Price, P. L. Permian to Jurassic palynostratigraphic nomenclature of the Bowen and Surat Basins. *In:* Green, P.M. (Ed.), The Surat and Bowen Basins, South-East Queensland. *Queensland Depart. Mines Energy*, pp. 137–178 (1997).

41. Collinson, J. W., Hammer, W. R., Askin, R. A., & Elliot, D. H. Permian–Triassic boundary in the central Transantarctic Mountains, Antarctica. *Geol. Soc. Am. Bull.* **118**, 747–763 (2006).

42. Lindström, S., & McLoughlin, S. Synchronous palynofloristic extinction andrecovery after the end-Permian event in the Prince Charles Mountains. Antarctica: implications for palynofloristic turnover across Gondwana. *Rev. Palaeobot. Palynol.* **145**, 89–122 (2007).

43. Vajda, V., & McLoughlin, S. Extinction and recovery patterns of the vegetation across the Cretaceous–Palaeogene boundary - a tool for unravelling the causes of the end-Permian mass extinction. *Rev. Palaeobot. Palynol.* **144**, 99–112 (2007).

44. Foster, C. B. Spore-pollen assemblages of the Bowen Basin, Queensland (Australia): their relationship to the Permian/Triassic boundary. *Rev. Palaeobot. Palynol.* 36, 165–183 (1982).

45. Barbolini, N., Bamford, M. K., & Rubidge, B. Radiometric dating demonstrates that Permian spore pollen zones of Australia and South Africa are diachronous. *Gond. Res.* **37**, 241–251 (2016).

46. Barbolini, N., Bamford, M. K., & Rubidge, B. A new approach to biostratigraphy in the Karoo retroarc foreland system: Utilising restricted-range palynomorphs and their first appearance datums for correlation. *J. Afr. Earth Sci.* **140**, 114–133 (2018).

47. Burgess, S. D., Bowring, S. A., & Shen, S.-Z. High-precision timeline for Earth's most severe extinction. *Proc. Natl. Acad. Sci. USA* **111**, 3316–3321 (2014).
