# Supplementary material for: The base of the Lystrosaurus Assemblage Zone, Karoo Basin, predates the end-Permian marine extinction
Source: Nat Commun. 2020 Mar 18;11:1428. doi: 10.1038/s41467-020-15243-7 (PMC7080820; doi:10.1038/s41467-020-15243-7)
Supplement: Supplementary file 3 — Reporting Summary [file 41467_2020_15243_MOESM3_ESM.pdf]

nature research

Corresponding author(s): Robert A. Gastaldo

Last updated by author(s): 25/2/2020

Reporting Summary

Nature Research wishes to improve the reproducibility of the work that we publish. This form provides structure for consistency and transparency in reporting. For further information on Nature Research policies, see Authors & Referees and the Editorial Policy Checklist .

Statistics

For all statistical analyses, confirm that the following items are present in the figure legend, table legend, main text, or Methods section.

n/a	Confirmed	
☐	☒	The exact sample size (n) for each experimental group/condition, given as a discrete number and unit of measurement
☐	☒	A statement on whether measurements were taken from distinct samples or whether the same sample was measured repeatedly
☒	☐	The statistical test(s) used AND whether they are one- or two-sided<br>*Only common tests should be described solely by name; describe more complex techniques in the Methods section.*
☒	☐	A description of all covariates tested
☒	☐	A description of any assumptions or corrections, such as tests of normality and adjustment for multiple comparisons
☒	☐	A full description of the statistical parameters including central tendency (e.g. means) or other basic estimates (e.g. regression coefficient) AND variation (e.g. standard deviation) or associated estimates of uncertainty (e.g. confidence intervals)
☒	☐	For null hypothesis testing, the test statistic (e.g. F, t, r) with confidence intervals, effect sizes, degrees of freedom and P value noted<br>*Give P values as exact values whenever suitable.*
☒	☐	For Bayesian analysis, information on the choice of priors and Markov chain Monte Carlo settings
☒	☐	For hierarchical and complex designs, identification of the appropriate level for tests and full reporting of outcomes
☒	☐	Estimates of effect sizes (e.g. Cohen's d, Pearson's r), indicating how they were calculated

Our web collection on statistics for biologists contains articles on many of the points above.

Software and code

Policy information about availability of computer code

Data collection	Micromass Isotope Ratio Software 1998, VG354 Release 3.969
Data analysis	Plotting of U-Pb data employed Isoplot, K.R. Ludwig, Berkeley Geochronology Center, June 2005, ver. 3.31 that include associated error ellipses for Pb/U ratios based on 2-sigma errors. Excel was used to average triplicate sample results for each horizon analyzed for Hg and TOC.

For manuscripts utilizing custom algorithms or software that are central to the research but not yet described in published literature, software must be made available to editors/reviewers. We strongly encourage code deposition in a community repository (e.g. GitHub). See the Nature Research guidelines for submitting code & software for further information.

Data

Policy information about availability of data

All manuscripts must include a data availability statement. This statement should provide the following information, where applicable:

- Accession codes, unique identifiers, or web links for publicly available datasets
- A list of figures that have associated raw data
- A description of any restrictions on data availability

The paleomagnetic and rock magnetic datasets generated during and/or analyzed during the current study are available on the MagIC database repository site (https://earthref.org/MagicIC) and on Paleomagnetism.org 2.0 (https://api.paleomagnetism.org). The authors declare that all additional data supporting the findings of this study are included in this published article (and its Supplementary Information, Figures, and Tables). Palynological slides are curated in the Museum of Paleontology, University of California, Berkeley, California, under the locality Nooitgedacht 68 – UCMP PA1378, with PA1378.01 (40.42m) and PA1378.02 (29.9m). Slides are N2990 P-1 and N2990 P-2, and N4042 P-1 and N4042 P-2. Specimen numbers: Plate 1, A–H: 398665–398672; Plate 2, A–X: 398673–398696.

Field-specific reporting

Please select the one below that is the best fit for your research. If you are not sure, read the appropriate sections before making your selection.

[ ] Life sciences [ ] Behavioural & social sciences [x] Ecological, evolutionary & environmental sciences

For a reference copy of the document with all sections, see nature.com/documents/nr-reporting-summary-flat.pdf

Ecological, evolutionary & environmental sciences study design

All studies must disclose on these points even when the disclosure is negative.

Study description

U-Pb CA ID-TIMS analyses of zircon grains in an ash fall depositand related concentrations of mercury in a lithostratigraphic and magnetostratigraphic context.

Research sample

Geochronology: Volcanic ash beds are uncommon in any stratigraphic succession and when found, are sampled as availability of exposure allows. A 1-cm-thick, light gray to white ash bed, exposed for less than 10 m of lateral extent, was excavated and sampled as conditions allowed. About ~800 grams of very fine-grained, light green ash from a ~1 cm-thick ash-fall layer was sampled with care taken to exclude material from adjacent beds and/or loose detritus that could contain zircon grains. Zircon grains average 200 micrometers in size, and yield of sufficient numbers for analyses would be possible from less than 1 kg of sediment. The layer is hosted in a ~0.5-m-thick, flat-lying, massive, reddish-gray siltstone unit on Farm Nooitgedacht 68, Bethulie District (S30.32616º, E025.93242º). For comparison of zircon populations, similar-sized samples of the massive red siltstone were collected from immediately above and below the ash bed.

Magnetostratigraphy: In general, only competent lithologies are sampled using a coring device. Due to the friable conditions of the weathered and exposed rock, we sampled weathered siltstone beds with a technique applied to unconsolidated sediment. Samples were taken 5 cm above and below the ~1 cm ash-fall bed sampled for geochronology, within the massive, ~0.5 m thick red siltstone interval. These were supplemented with ceramic-box samples obtained five meters below the ash horizon and eight meters above. Gastaldo et al. (2019) demonstrated that the zone over which the reported vertebrate turnover occurs is found in a reverse polarity chron on Bethel farm . In addition, Gastaldo et al. (2018) demonstrated that a short reverse polarity chron is found on farm Blaauwater in the Eastern Cape Province over which the vertebrate turnover may have occurred. Hence, (1) sampling the interval surrounding the ash bed was to determine its relationship to the established geochronological model for the latest Permian and (2) determine if the reported vertebrate turnover on Nooitgedacht occurred coincident with that on Bethel farm (30 km away) or represented a different point in time when the fossil assemblages were preserved.

Mercury: Samples of approximately 50 gm were taken at 5 cm intervals below, at, and above the ash fall deposit, and at 10 cm intervals across an interval identified by Botha-Brink et al. (2014) as the end-Permian extinction event, and equated to the critical interval in the marine realm. The marine crisis is associated with several Hg excursions and a similar pattern might be represented in the terrestrial record if both turnovers were coincident. Both TOC and Hg analyses are conducted on milligrams of sediment.

Sampling strategy

Zircons: About ~800 grams of very fine-grained, light green ash from a ~1 cm-thick ash-fall layer was sampled with care taken to exclude material from adjacent beds and/or loose detritus that could contain zircon grains.
Magnetostratigraphy: Siltstone/mudstone intervals are highly weathered and fragmentary. After cleaning off the exposure with non-magnetic implements, small (<0.7 cm) chips of thinly bedded siltstone were carefully placed into ceramic boxes (measuring 1.7 cm on a side; Beijing Eusci Technologies Ltd.) with ceramic lids, keeping each chip upright and roughly oriented with respect to Geographic north. Chips were removed using non-magnetic tweezers, and, if needed, they were shaped into appropriate size using non-magnetic (Cu-Be) tools. Glass wool or cotton was used to pack the chips in the ceramic boxes, to prevent movement or fragmentation during transport, and the boxes taped shut.
Mercury: see above

Data collection

Zircons: U and Pb were isolated from the zircon using 50 µl anion exchange columns using HCl, deposited onto outgassed rhenium filaments with silica gel(63), and analyzed with a VG354 mass spectrometer using a single Daly detector in pulse counting mode for Pb, and three Faraday cups in static analysis mode for U or Daly detector if the signal was <300KCps. Corrections to the 206Pb-238U ages for initial 230Th disequilibrium in the zircon have been made assuming a Th/U ratio in the magma of 4.2. All common Pb in each analysis was assigned the isotopic composition of procedural Pb blank. Dead time of the measuring system for Pb was 16 nanoseconds. The mass discrimination correction for the Daly detector is constant at 0.05% per atomic mass unit; the thermal mass fractionation correction for Pb was 0.10% per atomic mass unit (± 0.076%, 2σ); and the U thermal mass fractionation correction was measured and corrected within each measurement block for static runs. Amplifier gains and Daly characteristics were monitored using the SRM 982 Pb standard. Decay constants are those of Jaffey et al.(64).
Magnetics: In the laboratory, the glass wool or cotton was removed and the ceramic cubes filled with Zircar alumina cement, which is completely non-magnetic. The ceramic boxes were labeled using a soft aluminum rod, and then subjected to progressive thermal demagnetization using an ASC TD48 thermal demagnetization unit. Magnetizations were measured on a pulse-cooled DC SQUID 2G Enterprises magnetometer. Demagnetization data were inspected using orthogonal demagnetization diagrams(66) and directions of components of magnetization were determined using principal components analysis(67). The general dispersion (in declination) of magnetizations isolated in these materials is largely attributed to the nature of the sampling procedure, necessitated by the very fissile and friable nature of the hematitic siltstone.

Timing and spatial scale

Sampling of the ash occurred on two occasions. A pilot project sample was collected in 21 January 2017 to determine if zircon grains were present in the ash by RAG. These were sent to S. Kamo for disaggregation and inspection. Upon discovery that pristine zircon A second sampling of the horizon occurred on 15-17 January 2018 by RAG, SK, and JWG. The entire bed thickness was sampled over a

	lateral distance of 3 m. Zircon grains are not evenly distributed over the bed thickness, requiring complete sampling to obtain the most grains.
Data exclusions	No data are excluded.
Reproducibility	Reproducibility of U-Pb CA ID-TIMS age reproduced with 13 discrete zircon grains, as indicated on the concordia diagram.
Randomization	Randomization is not applicable to this study because zircon grains falling in ash are randomized during the volcanic event. Only euhedral, pristine zircon grains are analyzed due to the cost associated with CA ID-TIMS analyses.
Blinding	Subjects in an experiment often are given a placebo to determine what effects may occur in this control group and eliminate bias. Abiotic volcanic minerals .

Did the study involve field work? ☒ Yes ☐ No

Field work, collection and transport

Field conditions	The Karoo is a dry desert in summer (January 2017, 2018) and early fall (March 2019), with temperatures reaching 42 C during the day and 20C in the evening.
Location	Farm Nooitgedacht 68 (S 30.32530° , E 025.93132°), The Free State, South Africa, near Bethulie.
Access and import/export	Access to farm granted by owner.
Disturbance	The stratigraphic section was measured and small hand samples collected from exposed rock with no soil cover. Sample sizes were limited to avoid disturbance to the natural environment. No plants were harmed during field work.

Reporting for specific materials, systems and methods

We require information from authors about some types of materials, experimental systems and methods used in many studies. Here, indicate whether each material, system or method listed is relevant to your study. If you are not sure if a list item applies to your research, read the appropriate section before selecting a response.

Materials & experimental systems

n/a	Involved in the study	
☒	☐	Antibodies
☒	☐	Eukaryotic cell lines
☐	☒	Palaeontology
☒	☐	Animals and other organisms
☒	☐	Human research participants
☒	☐	Clinical data

Methods

n/a	Involved in the study	
☒	☐	ChIP-seq
☒	☐	Flow cytometry
☒	☐	MRI-based neuroimaging

Palaeontology

Specimen provenance	All samples, including those for palynological analyses, were obtained from our hand samples collected during sample measurement, for which no permission is required. We obtained permission to access the land from A.J. Griesel, owner. Palynological rich samples originate from laminated siltstone intervals on Farm Nooitgedacht 68, S 30.32530° , E 025.93132° .
Specimen deposition	Slides are curated in the Museum of Paleontology, University of California, Berkeley, California, under the locality Nooitgedacht 68 – UCMP PA1378, with PA1378.01 (40.42m) and PA1378.02 (29.9m). Preparations from horizons 30.35m and 30.42m yielded very low numbers spores and pollen from which it was not possible to evaluate either assemblage. Slides are N2990 P-1 and N2990 P-2, and N4042 P-1 and N4042 P-2. Specimen numbers: Plate 1, A–H: 398665–398672; Plate 2, A–X: 398673–398696.
Dating methods	About ~800 grams of very fine-grained, light green ash from a ~1 cm-thick ash-fall layer was sampled with care taken to exclude material from adjacent beds and/or loose detritus that could contain zircon grains. The layer is hosted in a ~0.5-m-thick, flat-lying, massive, reddish-gray siltstone unit on Farm Nooitgedacht 68, Bethulie District (S30.32616º, E025.93242º). For comparison of zircon populations, similar-sized samples of the massive red siltstone were collected from immediately above and below the ash bed.<br>The samples were disaggregated in a ring mill and a heavy mineral concentrate was produced on a Wilfley table. This was followed by standard mineral-separation procedures using magnetic (Isodynamic Frantz) and heavy liquid (methylene iodide) methods, the latter in small (~10 mL) centrifuge tubes.<br>U-Pb analysis was by isotope dilution-thermal ionization mass spectrometry methods on single chemically abraded zircon grains (CA-ID-TIMS) in the Jack Satterly Geochronology Laboratory of the Department of Earth Sciences at the University of Toronto. Prior to dissolution and analysis, zircon crystals were thermally annealed at 900º C for 48 hours to repair radiation damage in the crystal lattice. Subsequently, the grains were partially dissolved in ~0.1 ml ~50% hydrofluoric acid and ~0.020 ml of HNO3 at 195º C for 9 hours(61). Zircon grains were rinsed with 6N HCl followed by 8N HNO3 at room temperature prior to dissolution. A 205Pb-233-235U spike from the EARTHTIME Project or an in-house 205Pb-235U (ROM) spike was added to the Teflon dissolution capsules during sample loading. Zircon was dissolved using ~0.10 ml of concentrated HF acid and ~0.020 ml of 8N HNO3 at 200° C for 5 days, then dried to a precipitate and re-dissolved in ~0.15 ml of 3N HCl at 200 C overnight(62). U and Pb were isolated from the zircon using 50 µl anion exchange columns using HCl, deposited onto outgassed rhenium filaments with silica gel(63), and analyzed with a VG354 mass spectrometer using a single Daly detector in pulse counting mode for Pb, and three Faraday cups in static analysis mode for U or Daly detector if the signal was <300KCps. Corrections to the 206Pb-238U ages for initial 230Th disequilibrium in the zircon have been made assuming a Th/U ratio in the magma of 4.2. All common Pb in each analysis was assigned the isotopic composition of procedural Pb blank. Dead time of the measuring system for Pb was 16 nanoseconds. The mass discrimination correction for the Daly detector is constant at 0.05% per atomic mass unit; the thermal mass fractionation correction for Pb was 0.10% per atomic mass unit (± 0.076%, 2σ); and the U thermal mass fractionation correction was measured and corrected within each measurement block for static runs. Amplifier gains and Daly characteristics were monitored using the SRM 982 Pb standard. Decay constants are those of Jaffey et al.(64). Age errors quoted in the text and table, and error ellipses in the concordia diagram and weighted mean age plot are given at the 95% confidence interval. Plotting of U-Pb data employed Isoplot 3.76(65).

☒ Tick this box to confirm that the raw and calibrated dates are available in the paper or in Supplementary Information.
